# Platform-Based Patient-Clinician Digital Health Interventions for Care Transitions: Protocol for a Scoping Review

**DOI:** 10.2196/42056

**Published:** 2023-04-05

**Authors:** Chantal Backman, Steve Papp, Anne Harley, Aurelie Tonjock Kolle, Sarah Visintini, Soha Shah, Randa Berdusco, Stéphane Poitras, Paul E Beaulé, Veronique French-Merkley

**Affiliations:** 1 University of Ottawa Ottawa, ON Canada; 2 The Ottawa Hospital Ottawa, ON Canada; 3 Bruyère Continuing Care Ottawa, ON Canada; 4 Alberta Health Services Calgary, AB Canada

**Keywords:** care transitions, digital health, targeted patient/client communication, scoping review protocol, patient care, patient support, hospital-to-home transition, hospital discharge, implementation, barriers

## Abstract

**Background:**

With the increased adoption of technology, the use of digital health interventions in health care settings has increased. Patient-clinician digital health interventions have the potential to improve patient care, especially during important transitions between hospital and home. Digital health interventions can provide support to patients during these transitions, thereby leading to better patient outcomes.

**Objective:**

This scoping review aims to explore the available literature, specifically (1) to examine the impact of platform-based digital health interventions focused on care transitions on patient outcomes, and (2) to identify the barriers to and enablers for the uptake and implementation of these digital health interventions.

**Methods:**

This protocol was developed based on Arksey and O’Malley’s, Levac and colleagues’, and JBI scoping review methodologies, and it has been reported according to the PRISMA-ScR (Preferred Reporting Items for Systematic Reviews and Meta-Analyses Statement for the Scoping Reviews) format. The search strategies were developed for 4 databases: MEDLINE, CINAHL, EMBASE, and the Cochrane Central Register of Controlled Trials by using key words such as “hospital to home transition” and “platform-based digital health.” Studies involving patients 16 years or older that used a platform-based digital health intervention during their hospital to home transition will be included in this review. Two reviewers will independently screen articles for eligibility by using a 2-stage process (ie, title and abstract screening and full-text screening). We expect to refine the eligibility criteria during the title and abstract screening process as we anticipate retrieving a significant number of articles. In addition, we will also perform a targeted search of the grey literature, as well as data extraction. Data analysis will consist of a narrative and descriptive synthesis.

**Results:**

The review is expected to identify research gaps that will inform the development of future patient-clinician digital health interventions. We have identified a total of 8333 articles. Screening began in September 2022, and data extraction is expected to commence in February 2023 and end by April 2023. Data analyses and final results will be submitted to a peer-reviewed journal in August 2023.

**Conclusions:**

We expect to find a wide variety of postcare interventions, some gaps in the quality of research evidence, as well as a lack of detailed information on digital health interventions.

**International Registered Report Identifier (IRRID):**

PRR1-10.2196/42056

## Introduction

Patient-clinician digital health interventions have been described by the World Health Organization as “targeted patient/client communication” [1] and have become increasingly popular in health care settings [2]. Such interventions can support patients and caregivers in navigating a complex health system by utilizing information technologies to facilitate communication between parties [1,3]. Digital health interventions that use platform-based technology to facilitate communication and information-sharing between patients and clinicians have the potential to improve care coordination and outcomes during care transitions.

Specifically, asynchronous digital platforms such as web-based or mobile applications contain information that is shared or stored between patients and clinicians. These digital platforms often include patient information and education for discharge planning, follow-up appointments and services, patient empowerment and engagement, and health status updates [1]. In a recent scoping review by Zhang and colleagues [4], the role of digital health interventions for patients with hip fractures was examined. Results of the scoping review indicated that digital health interventions were generally used to support health care providers in the care of such patients. In another systematic review by Free and colleagues [3], digital health interventions were shown to have increased the engagement of patients in their health care journey and a corresponding reduction in hospital admissions.

Engaging patients in their care through digital health interventions is possible given the widespread use of internet-based personal communication devices [5] and the effective use of such interventions with patients and their clinicians. Although asynchronous digital platforms can sometimes be challenging, especially in relation to technological issues [6], successful use of these platforms has been described with patients living with diabetes [7,8], cardiovascular disease [9,10], chronic obstructive pulmonary disease [11], and patients recovering post surgery [12-14].

The multi-service and multi-sector coordination of care after an acute event or diagnosis can be complicated for patients and caregivers to navigate. After acute care admission, patients often receive inadequate information about discharge instructions [15], whereas others have unanswered questions [16]. Some studies have described how patients may experience poor retention of verbal instructions during post-discharge periods [17,18], and how effective education and written discharge instructions may help improve a patient’s understanding and facilitate transition from hospital to home [19-23]. A critical gap that has been identified is poor communication between clinicians and patients and their caregivers [24,25]. Digital health interventions can provide opportunities for ongoing support from clinicians to patients and their caregivers [12], which can improve the overall level of communication and lead to better adherence to care instructions [26].

The emerging research highlighting the benefits of patient engagement [27] reinforces the importance of developing patient-centered discharge planning processes [28]. This may include education for patients and their caregivers regarding their health conditions, medication, and common symptoms; a mechanism for exchanging questions and obtaining feedback from patients; and guidance on when to seek appropriate medical care or follow-ups. Using integrated [29] and holistic patient and caregiver interventions [19-23,30], and digital platforms [31] in particular, can help minimize avoidable readmissions and other challenges with the transition of patients from hospital to their homes. Digital health interventions should be inclusive and consider unique patient characteristics such as age, disability, and level of cognition to optimize success [32]. The usability of digital health interventions for patients and clinicians is also crucial for their successful uptake [33].

Despite the widespread development and use of information technology [5], the desire of patients and caregivers for readily available information [34], and the need for better engaging patients in their care process to optimize patient outcomes, only limited research has examined the specific platform-based patient-clinician digital health interventions that are in use for discharge and care transitions.

Our goal is to explore the available literature, specifically (1) to examine the impact of platform-based digital health interventions specific to care transitions on patient outcomes, and (2) to identify the barriers to and enablers for the uptake and implementation of these digital health interventions. The findings from this work would inform future work on the “MyPath to Home” digital health intervention [35].

## Methods

### Protocol Design

This protocol was developed based on Arksey and O’Malley’s [36] methodology, elaborated by Levac and colleagues [37] and JBI Manual for Evidence Synthesis [38], which involves the following stages: (1) identify the research question; (2) identify relevant studies: (3) select studies; (4) chart the data; and (5) collate, synthesize, and report results. For this protocol, we also followed the PRISMA-ScR (Preferred Reporting Items for Systematic Reviews and Meta-Analyses Statement for the Scoping Reviews) reporting format [39].

### Stage 1: Identify the Research Question

The research questions guiding this scoping review are as follows: 

1. What is the impact of platform-based patient-clinician digital health interventions used for discharge or care transitions on patient outcomes?
2. What are the barriers to and enablers for the uptake and implementation of these interventions for clinicians and patients transitioning from hospital to home?

### Stage 2: Identify Relevant Studies

A comprehensive search strategy will be developed by an information specialist relevant to MEDLINE, CINAHL, Embase, and the Cochrane Central Register for Controlled Trials. The MEDLINE strategy will be peer-reviewed by another information specialist according to the PRESS (Peer Review of Electronic Search Strategies) guidelines [40]. The search strategies are available in Multimedia Appendix 1.

Once the searches have been completed, the results will be exported to Covidence [41] for storage, removal of duplicates, and screening. In addition, we will conduct a manual search of the references from the included studies and a targeted grey literature search of ClinicalTrials.gov [42] and the International Clinical Trials Registry Platform Search Portal [43], as well as key digital health technology websites.

### Stage 3: Select Studies

Preliminary inclusion criteria are defined following the Population, Concept, Context (PCC) format:

- Population: Patients (16 years or older) recruited before hospital discharge;
- Concept: Postcare interventions (eg, self-care, symptom monitoring, pain control and management, mobilization, follow-up appointments, ability to ask questions) using any asynchronous digital platforms such as web-based or mobile applications where information is shared or stored between patients and clinicians; and
- Context: Discharge episode from hospital to the community.

We will include primary studies (ie, randomized controlled trials [RCTs], quasi-experimental studies, pilot or feasibility studies, observational studies [case-control, cohort, cross-sectional, and descriptive studies], and qualitative studies) published between 2012 and 2022. Preliminary exclusion criteria will include: (1) pediatric, newborn care, mental health, and cancer populations; (2) synchronous non–platform-based digital health care interventions (eg, chat platform, wearable devices, prosthetics, robotics, medical imaging technology such as x-rays and ultrasounds, interventions only using a standard telephone); and (3) nonprimary studies (eg, commentaries, editorials, study protocols, and policy briefs).

Study selection will be performed independently by two reviewers (AF and RP) in two steps: (1) screening of abstracts and titles and (2) screening of full-text articles. Any disagreement in screening decisions will be resolved through discussion among the reviewers or by a third reviewer (CB). A list of the excluded studies from the full-text review will be reported using the PRISMA-ScR reporting format [39].

### Stage 4: Chart the Data

Two reviewers (AF and RP) will independently pilot the data extraction by using a form designed in Microsoft Excel (Microsoft Corp.). These reviewers will then independently extract the data from the eligible studies. Data extracted will include authors, year of publication, study location, purpose, study design, theoretical approach, study population, description of intervention (and comparator), data analysis, and study results or outcomes (eg, patient outcomes, barriers, and enablers). To describe the interventions, we will follow recommendations from the Template for Intervention Description and Replication checklist [44], comprising brief name of the intervention; why; materials used; procedures; who provided; how; where; when and how much; tailoring; modifications; how well (planned); and how well (implemented).

### Stage 5: Collate, Synthesize, and Report Results

We will conduct a narrative data synthesis, including quantitative descriptive analyses for quantitative studies and thematic analyses for qualitative studies. We will group data into tables by type of intervention, and by outcomes or findings.

For the barriers and enablers analysis, we will group the findings using the 14 domains of the Theoretical Domain Framework: knowledge, skills, social or professional role and identity, beliefs about capabilities, optimism, beliefs about consequences, reinforcement, intentions, goals, memory, attention, and decision processes, environmental context and resources, social influences, emotion, and behavioral regulation [45-47]. Specifically, the analysis will follow 3 steps. First, two reviewers (AF and RP) will independently map the findings to each of the Theoretical Domain Framework domains. Second, similar findings will be themed and grouped together. Third, each theme will be coded as either a barrier or an enabler. We will report the top barriers and enablers using descriptive statistics (eg, frequency and percentage for each barrier and enabler). Disagreements will be discussed and resolved by the two reviewers (AF and RP) or by consulting a third reviewer (CB).

## Results

The scoping review began in September 2022 and is expected to be completed in August 2023. We have identified a total of 8333 articles. The screening process took place between September 2022 and February 2023, and data extraction is expected to be completed in April 2023. Data analyses and final results will be submitted to a peer-reviewed journal in August 2023.

Through this review, we intend to provide an overview of the current state of research on platform-based, patient-clinician digital health interventions for care transitions, including a discussion of the different types of platforms and interventions that have been studied, the evidence for their effectiveness, and the barriers to and enablers for future research in this area.

## Discussion

Overall, we will provide a comprehensive picture of the current state of knowledge on the use of digital health interventions in care transitions, and the potential for these interventions to improve patient outcomes. We expect to find a wide variety of postcare interventions, some gaps in the quality of the research evidence, as well as a lack of detailed information on the digital health interventions. Our results will include an overview of patient-clinician digital health interventions for discharge or care transitions, as well as barriers to and enablers for their implementation. In addition, this review is expected to identify research gaps that will inform the development of future patient-clinician digital health interventions.

Our review has several strengths and limitations. We designed an in-depth a priori protocol. The search strategy was developed and peer-reviewed by a research librarian with extensive knowledge in scoping and systematic reviews.  However, we anticipate that the digital health interventions will be heterogeneous, making it more difficult to make comparisons and draw specific conclusions.

The results of our review would be beneficial to various stakeholders such as researchers, clinicians, administrators, and policymakers. We will disseminate our findings through publication in a peer-reviewed journal as well as by presenting our results at a scientific conference.

## Appendix

Multimedia Appendix 1 Search strategies.

## Abbreviations

PCC: Population, Concept, Context
PRESS: Peer Review of Electronic Search Strategies
PRISMA-ScR: Preferred Reporting Items for Systematic Reviews and Meta-Analyses Statement for the Scoping Reviews
