# Supplementary material for: Platform-Based Patient-Clinician Digital Health Interventions for Care Transitions: Protocol for a Scoping Review
Source: JMIR Res Protoc. 2023 Apr 5;12:e42056. doi: 10.2196/42056 (PMC10131754; doi:10.2196/42056)
Supplement: Multimedia Appendix 1 [file resprot_v12i1e42056_app1.docx]

## Table 1. Medline Search

| **#** | **Searches** |
| --- | --- |
| 1 | hospital to home transition/ or patient discharge/ or Transitional Care/ |
| 2 | (discharg* or post-hospital* or posthospital* or after-hospital* or Postoperat* or post-operat* or Postsurger* or post-surger* or Postsurgical* or post-surgical* or after surger* or after operat*).ti,ab,kf. |
| 3 | (transition* adj4 (home? or house? or community or residence* or hospital* or care or pathway* or protocol* or healthcare or navigat*)).ti,ab,kf. |
| 4 | (home adj4 (return* or transition* or reintegrat* or re-integrat* or follow-up or followup)).ti,ab,kf. |
| 5 | or/1-4 |
| 6 | Computers/ or Internet/ or Internet-Based Intervention/ or Therapy, Computer-Assisted/ or Mobile Applications/ or Web Browser/ |
| 7 | (mhealth or m-health or "mobile health" or ehealth or e-health or digital health or ePRO).ti,ab,kf. |
| 8 | ((web or website? or web-based or internet* or online or patient* or mobile* or computer* or PC or tablet* or digital* or digiti* or electronic*) adj3 (application* or app? or portal* or platform* or hub? or dashboard*)).ti,ab,kf. |
| 9 | or/6-8 |
| 10 | 5 and 9 |
| 11 | limit 10 to yr="2012 -Current" |

## Table 2. Embase Search

| **#** | **Searches** |
| --- | --- |
| 1 | exp *hospital discharge/ or *transitional care/ |
| 2 | (discharg* or post-hospital* or posthospital* or after-hospital* or Postoperat* or post-operat* or Postsurger* or post-surger* or Postsurgical* or post-surgical* or after surger* or after operat*).ti,ab,kf. |
| 3 | (transition* adj4 (home? or house? or community or residence* or hospital* or care or pathway* or protocol* or healthcare or navigat*)).ti,ab,kf. |
| 4 | (home adj4 (return* or transition* or reintegrat* or re-integrat* or follow-up or followup)).ti,ab,kf. |
| 5 | or/1-4 |
| 6 | exp *personal computer/ or *computer/ or *internet/ or *web-based intervention/ or exp *computer assisted therapy/ or exp *self-care software/ or exp *mobile application/ or *web browser/ |
| 7 | (mhealth or m-health or "mobile health" or ehealth or e-health or digital health or ePRO).ti,ab,kf. |
| 8 | ((web or website? or web-based or internet* or online or patient* or mobile* or computer* or PC or tablet* or digital* or digiti* or electronic*) adj3 (application* or app? or portal* or platform* or hub? or dashboard*)).ti,ab,kf. |
| 9 | or/6-8 |
| 10 | 5 and 9 |
| 11 | limit 10 to yr="2012 -Current" |

## Table 3. Cochrane Central Register of Controlled Trials Search

| **#** | **Searches** |
| --- | --- |
| 1 | hospital to home transition/ or patient discharge/ or Transitional Care/ |
| 2 | (discharg* or post-hospital* or posthospital* or after-hospital* or Postoperat* or post-operat* or Postsurger* or post-surger* or Postsurgical* or post-surgical* or after surger* or after operat*).ti,ab,kw. |
| 3 | (transition* adj4 (home? or house? or community or residence* or hospital* or care or pathway* or protocol* or healthcare or navigat*)).ti,ab,kw. |
| 4 | (home adj4 (return* or transition* or reintegrat* or re-integrat* or follow-up or followup)).ti,ab,kw. |
| 5 | or/1-4 |
| 6 | Computers/ or Internet/ or Internet-Based Intervention/ or Therapy, Computer-Assisted/ or Mobile Applications/ or Web Browser/ |
| 7 | (mhealth or m-health or "mobile health" or ehealth or e-health or digital health or ePRO).ti,ab,kw. |
| 8 | ((web or website? or web-based or internet* or online or patient* or mobile* or computer* or PC or tablet* or digital* or digiti* or electronic*) adj3 (application* or app? or portal* or platform* or hub? or dashboard*)).ti,ab,kw. |
| 9 | or/6-8 |
| 10 | 5 and 9 |
| 11 | limit 10 to yr="2012 -Current" |

## Table 4. CINAHL Search

| **#** | **Query** | **Limiters/Expanders** | **Last Run Via** |
| --- | --- | --- | --- |
| S16 | S7 AND S14 | Limiters - Published Date: 20110101-20221231 Expanders - Apply related words; Apply equivalent subjects Search modes - Boolean/Phrase | Interface - EBSCOhost Research Databases Search Screen - Advanced Search Database - CINAHL |
| S15 | S7 AND S14 | Expanders - Apply related words; Apply equivalent subjects Search modes - Boolean/Phrase | Interface - EBSCOhost Research Databases Search Screen - Advanced Search Database - CINAHL |
| S14 | S8 OR S9 OR S10 OR S11 OR S12 OR S13 | Expanders - Apply related words; Apply equivalent subjects Search modes - Boolean/Phrase | Interface - EBSCOhost Research Databases Search Screen - Advanced Search Database - CINAHL |
| S13 | TI ( ((web or website# or web-based or internet* or online or patient* or mobile* or computer* or PC or tablet* or digital* or digiti* or electronic*) N3 (application* or app# or portal* or platform* or hub# or dashboard*)) ) OR AB ( ((web or website# or web-based or internet* or online or patient* or mobile* or computer* or PC or tablet* or digital* or digiti* or electronic*) N3 (application* or app# or portal* or platform* or hub# or dashboard*)) ) | Expanders - Apply related words; Apply equivalent subjects Search modes - Boolean/Phrase | Interface - EBSCOhost Research Databases Search Screen - Advanced Search Database - CINAHL |
| S12 | TI ( (mhealth or m-health or "mobile health" or ehealth or e-health or digital health or ePRO) ) OR AB ( (mhealth or m-health or "mobile health" or ehealth or e-health or digital health or ePRO) ) | Expanders - Apply related words; Apply equivalent subjects Search modes - Boolean/Phrase | Interface - EBSCOhost Research Databases Search Screen - Advanced Search Database - CINAHL |
| S11 | (MH "Mobile Applications") OR (MH "Web Browsers") | Expanders - Apply related words; Apply equivalent subjects Search modes - Boolean/Phrase | Interface - EBSCOhost Research Databases Search Screen - Advanced Search Database - CINAHL |
| S10 | (MH "Therapy, Computer Assisted") OR (MH "Drug Therapy, Computer Assisted") | Expanders - Apply related words; Apply equivalent subjects Search modes - Boolean/Phrase | Interface - EBSCOhost Research Databases Search Screen - Advanced Search Database - CINAHL |
| S9 | (MH "Internet") OR (MH "Internet-Based Intervention") | Expanders - Apply related words; Apply equivalent subjects Search modes - Boolean/Phrase | Interface - EBSCOhost Research Databases Search Screen - Advanced Search Database - CINAHL |
| S8 | (MH "Computers, Portable+") | Expanders - Apply related words; Apply equivalent subjects Search modes - Boolean/Phrase | Interface - EBSCOhost Research Databases Search Screen - Advanced Search Database - CINAHL |
| S7 | S1 OR S2 OR S3 OR S4 OR S5 OR S6 | Expanders - Apply related words; Apply equivalent subjects Search modes - Boolean/Phrase | Interface - EBSCOhost Research Databases Search Screen - Advanced Search Database - CINAHL |
| S6 | TI ( (home N4 (return* or transition* or reintegrat* or re-integrat* or follow-up or followup)) ) OR AB ( (home N4 (return* or transition* or reintegrat* or re-integrat* or follow-up or followup)) ) | Expanders - Apply related words; Apply equivalent subjects Search modes - Boolean/Phrase | Interface - EBSCOhost Research Databases Search Screen - Advanced Search Database - CINAHL |
| S5 | TI ( (transition* N4 (home# or house# or community or residence* or hospital* or care or pathway* or protocol* or healthcare or navigat*)) ) OR AB ( (transition* N4 (home# or house# or community or residence* or hospital* or care or pathway* or protocol* or healthcare or navigat*)) ) | Expanders - Apply related words; Apply equivalent subjects Search modes - Boolean/Phrase | Interface - EBSCOhost Research Databases Search Screen - Advanced Search Database - CINAHL |
| S4 | TI ( (discharg* or post-hospital* or posthospital* or after-hospital* or Postoperat* or post-operat* or Postsurger* or post-surger* or Postsurgical* or post-surgical* or after surger* or after operat*) ) OR AB ( (discharg* or post-hospital* or posthospital* or after-hospital* or Postoperat* or post-operat* or Postsurger* or post-surger* or Postsurgical* or post-surgical* or after surger* or after operat*) ) | Expanders - Apply related words; Apply equivalent subjects Search modes - Boolean/Phrase | Interface - EBSCOhost Research Databases Search Screen - Advanced Search Database - CINAHL |
| S3 | (MH "Transitional Care") | Expanders - Apply related words; Apply equivalent subjects Search modes - Boolean/Phrase | Interface - EBSCOhost Research Databases Search Screen - Advanced Search Database - CINAHL |
| S2 | (MH "Patient Discharge") OR (MH "Patient Discharge Education") | Expanders - Apply related words; Apply equivalent subjects Search modes - Boolean/Phrase | Interface - EBSCOhost Research Databases Search Screen - Advanced Search Database - CINAHL |
| S1 | (MH "Hospital to Home Transition") | Expanders - Apply related words; Apply equivalent subjects Search modes - Boolean/Phrase | Interface - EBSCOhost Research Databases Search Screen - Advanced Search Database - CINAHL |
